# Supplementary material for: N20D/N116E Combined Mutant Downward Shifted the pH Optimum of Bacillus subtilis NADH Oxidase
Source: Biology (Basel). 2023 Mar 30;12(4):522. doi: 10.3390/biology12040522 (PMC10135872; doi:10.3390/biology12040522)
Supplement: Supplementary file 1 [file biology-12-00522-s001.zip › biology-2253943-supplementary.pdf]

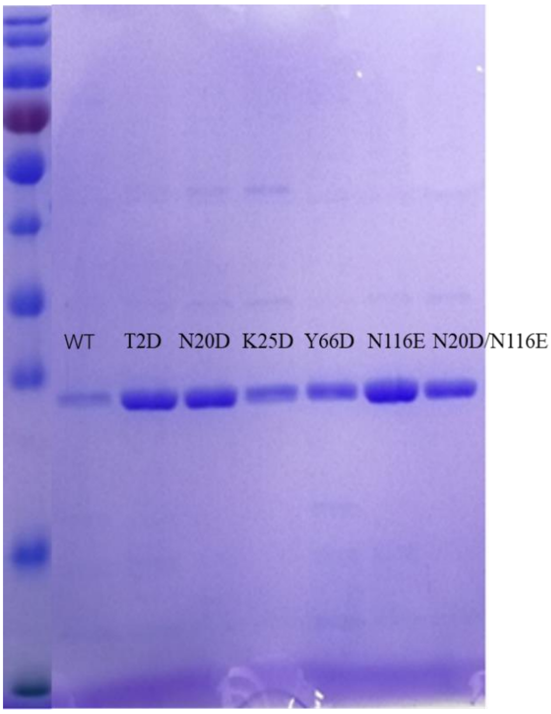

Figure S1 The gel of the purified BsNox and its variants

The purified proteins were examined by SDS-PAGE under denaturing conditions, using the prestained marker (Takara, Japan) as the reference.

Table S1 The bacterial strains, plasmids, and primers used in this study

Strains, plasmids, and primers	Relevant characteristic or sequence	Source
**Strains**		
Escherichia coli BL21(DE3)	Host	Laboratory stock
*Bacillus subtilis*168	For *BsNox* gene cloning	Laboratory stock
BL21/pETduet-1-$Bsgdh_{K218D}$	*E. coli* BL21 with pETduet-1-$Bsgdh_{K218D}$	Laboratory stock
BL21/pETduet-1-$BsNox_{N20D+N116E}$	*E. coli* BL21 with pETduet-1-B$sNox_{N20D+N116E}$	This study
**Plasmids**		
pETduet-1	Expression vector	Laboratory stock
pETduet-1-$BsNox_{N20D+N116E}$	pETduet-1 with $BsNOX_{N20D+N116E}$	This study
**Primers**		
BsNOX-F	AAGAAGGAGATATACATATGATGACGAATACTCTGGATGTTT TAAAAGCA (*Nde*I)	
BsNOX-R	GTTTCTTTACCAGACTCGAGTTAGTGGTGATGATGGTGATGC AGCCAAGTTGATACTTTTGAAAGCG (*Xho*I)	
BsNOX-T2D	CATATGATGGACAATACTCTGGATGTTTTAAAAG	
BsNOX-T2D	CAGAGTATTGTCCATCATATGTATATCTCCTTC	

BsNOX-K25D	GATCTCTGACGAGGAGCTGACT
BsNOX-K25D	CTCCTCGTCAGAGATCGGGG
BsNOX-Y66E	TGTAGCGGAAAATCAAAAACAAATCGT
BsNOX-Y66E	TTGATTTTCCGCTACAGGAAGAAGC
BsNOX-Y66D	TGTAGCGGACAATCAAAAACAAATCG
BsNOX-Y66D	TTGATTGTCCGCTACAGGAAGAAG
BsNOX-Q68E	AGCGTATAATGAAAAACAAATCGTTGAG
BsNOX-Q68E	CGATTTGTTTTTCATTATACGCTACAGGA
BsNOX-K85D	GCGATTTAGACGCAAATGAAAACG
BsNOX-K85D	CATTTGCGTCTAAATCGCCTAAAATG
BsNOX-Q114E	CTCGGCGAAATCAACGGTG
BsNOX-Q114E	GTTGATTTCGCCGAGCAATGT
BsNOX-A118D	AACGGTGACTACCAAAGCGA
BsNOX-A118D	TTGGTAGTCACCGTTGATTTGGC
BsNOX-Y119E	GGTGCTGAACAAAGCGAGC
BsNOX-Y119E	GCTTTGTTCAGCACCGTTG
BsNOX-I168D	TTTGATGACAGTGAGCGCTATGT
BsNOX-I168D	GCTCACTGTCATCAAATTGCTTTTG
BsNOX-R171E	AGTGAGGAATATGTTCCGGTTATGC
BsNOX-R171E	AACCGGAACATATTCCTCACTGATATC
BsNOX-H188E	CCTGCGGAACAAAGCAACC
BsNOX-H188E	GCTTTGTTCCGCAGGCTTC
BsNOX-Q189D	CGCATGACAGCAACCGTCT
BsNOX-Q189D	TTGCTGTCATGCGCAGGC
BsNOX-K197D	GCTTTCAGACGTATCAACTTGGC
BsNOX-K197D	GTTGATACGTCTGAAAGCGGC
BsNOX-N20D	TGATACAGACGCCCCGATCTCTAA
BsNOX-N20D	GGGGCGTCTGTATCATATTCCT
BsNOX-N116E	GCCAAATCGAAGGTGCTTACC
BsNOX-N116E	GCACCTTCGATTTGGCCGA
